# Acceptability of provider-initiated HIV testing as an intervention for prevention of mother to child transmission of HIV and associated factors among pregnant women attending at Public Health Facilities in Assosa town, Northwest Ethiopia

**DOI:** 10.1186/s13104-015-1652-4

**Published:** 2015-11-09

**Authors:** Solomon Abtew, Worku Awoke, Anemaw Asrat

**Affiliations:** College of Medicine and Health Sciences, Bahir Dar University, P.O. Box 693, Bahir Dar, Ethiopia

**Keywords:** Pregnant women, Provider-initiated HIV testing and counselling

## Abstract

**Background:**

Despite more efforts for prevention of mother to child HIV transmission, still there are problems with provider-initiated HIV testing. This study was done to assess the acceptance rate of provider-initiated HIV testing among antenatal care attendants and its associated factors.

**Methods:**

Institutions based cross sectional study with a sample size of 398 was conducted from February to March 2014 in two health facilities in Assosa town. Proportional allocation of the sample size of health facilities followed by systematic sampling method was done; data were collected using an interviewer administered questionnaire. Bivariate and multivariate regression analysis was employed using SPSS version 20.

**Results:**

A total of 386 pregnant women participated with response rate 97 % and 312 (80.8 %) of them accepted provider-initiated HIV testing. The odds of acceptance of provider-initiated HIV testing was higher among rural residents (AOR 4.04; 95 % CI 1.24–13.11) than urban. It was also higher among students (AOR 6.00; 95 % CI 1.45–24.75), merchants (AOR 4.43; 95 % CI 1.18–16.68) and employed women (AOR 2.15; 95 % CI 1.08–4.30) than housewives. Pregnant women who had no stigmatized attitude towards people living with HIV/AIDS were more likely to accept testing (AOR 3.54; 95 % CI 1.23–10.16) than who had a strong stigmatized attitude. In addition, those who planned to disclose their test results from their husbands were higher odd of acceptance (AOR 14.85; 95 % CI 4.60–47.94) than who secreted.

**Conclusion:**

Acceptance of provider-initiated HIV testing among pregnant women attending for antenatal care services was relatively high. Mothers from urban residence, occupational satus being housewives, stigmatization and not having a plan to disclose the status of test results were negatively affect the acceptance of provider-initiated HIV testing. During counselling sessions, antenatal care providers should focus on barriers of provider-initiated HIV testing such as residence, occupational status, stigmatized attitudes and disclosure status of results of HIV tests.

## Background

Worldwide rapid expansion of HIV/AIDS has a profound impact on the health sector and the socioeconomic development in general. Globally a total of 34 million people were living with HIV and of these about 69 % were in Sub Saharan Africa. In 2011, around 330,000 children acquired HIV infection mainly from mother to child transmission and of these more than 90 % were in sub-Saharan Africa [1].

In year 2011, the prevalence of HIV in Ethiopia was 1.5 % [2]. In year 2012 only, an estimated 37, 605 children in ager group of 0–4 years were HIV positive, more than 759, 268 people were living with HIV, around 20,158 newly infected in year 2012 and a total of 41,444 AIDS cases died. Of the total people living with HIV/AIDS (PLWHA), more than 22,057 were pregnant women [3]. The prevalence of HIV in Benshangul Gumuz Regional state, Ethiopia was 1.7 % [2] and ANC-based HIV prevalence among pregnant women and Assosa hospital was 17.8 and 7.6 % respectively [4].

The most significant source of HIV infection in children and infants are transmission of HIV from mother to child. Without interventions, the risk of transmission varies and ranging from 5–10 % during pregnancy, 10–20 % during labour/delivery and 10–20 % through mixed infant feeding [5, 6].

To address HIV/AIDS epidemics, prevention of mother to child transmission (PMTCT) of HIV becomes a priority for many developing country governments and agencies. PMTCT is a commonly used intervention designed to reduce the risk of mother to child transmission of HIV (MTCT). Among these, HIV testing and counselling (HTC) is a critical component and gate way for all pregnant mothers to learn whether they are infected, helped to understand the implications of their HIV status and make informed choices for the future to reduce morbidity, mortality and HIV transmission [7, 8].

Early detection of maternal HIV infection in pregnancy focused on voluntary counselling and testing (VCT) as the primary means of providing testing and encouraging people to become aware of their HIV status [9]. However Population surveys conducted in 2007–2009 in low- and middle-income countries showed that the median percentage of people living with HIV who know their status was estimated below 40 % [10]. Evidence showed that VCT coverage for target populations was low in Ethiopia [11].

Thus, World Health Organization (WHO) introduced provider-initiated HIV testing and counselling (PITC) approach and subsequently, the revised version of the Ethiopian PMTCT guideline adopted in 2007 recommends that provider-initiated HIV testing and counselling (PITC) as a routine care for pregnant women in antenatal care (ANC) clinics to decrease MTCT of HIV/AIDS [12, 13].

Even with great efforts have been done in Ethiopia after the introduction to PITC, only 11 % pregnant women tested for HIV in 2011 during their ANC visits [14]. Meanwhile limited studies conducted in other regions of Ethiopia, the acceptance rate and associated factors of PITC among pregnant women vary from one context to another [15–18] as well as some of the associated factors were contradicted. In addition the study area categorized as among the developing regions of Ethiopia and there was no study conducted about acceptance rate of PITC as an intervention of PMTCT among ANC attendants. Therefore, this study was done to assess acceptability of PITC as an intervention for prevention of mother to child transmission of HIV and to identify the associated factors that will help health planners and mangers for fair decision making process in resource limited setting in Ethiopia.

## Methods

Health facility based cross sectional study was conducted from February to March, 2014 in Assosa town. Assosa town is the capital city of Benshangul Gumuz National, Regional State, located Northwest of Ethiopia at 680 km from Addis Ababa. According to the 2007 central statistics agency report of Ethiopia, a total population of the town was 24,214 (12,463 were males and 11,751 were females) [19].

Administratively, the town is structured into four urban kebeles. It has one general hospital and one health centre. Both health facilities currently provide ANC, PMTCT and PITC for pregnant mothers. PITC is given under informed consent to all pregnant mothers attending to the facilities based on national testing strategy guideline [13]. Based on the record review prior to data collection, the average flow of pregnant mothers in the hospital and the heal center were 450 and 250 for all ANC visits (ANC visit one up to four) per month, respectively.

The source population was all pregnant women attended ANC in the health facilities. The study population was 15–49 years’ old pregnant women who attended ANC services during the study period. The study units were 15–49 years’ old pregnant women who attended ANC services during the study period in Assosa town public health facilities and who selected by sampling procedures. Pregnant women, Women who were critically sick, unable to hear, or had another disability that impaired their ability to communicate were excluded.

The sample size was calculated using single population proportion formula considering the following assumptions; 95 % confidence interval with the corresponding value 1.96, P = 56 % (proportion of pregnant women had comprehensive knowledge on HIV/AIDS [15]), the margin of error 0.05 and with the addition of 5 % non-response rate, the total sample size became 398.

The sample size was proportionally allocated to the two health facilities based on the flow of ANC clients. After routine ANC visits, participants were approached for consent using a systematic random sampling method. During selection, the first pregnant woman was selected randomly by lottery method and then every other ANC follower was included in the study after obtaining their consent. Finally, data was collected using interviewer administered questionnaires until the required sample size was achieved.

### Operational definitions

*Acceptors* Antenatal care attendants who accepted/tested to provider-initiated HIV counselling as an intervention for PMTCT during their ANC follow up.

*Knowledge* HIV/AIDS knowledge index was built from the answers to 13 questions: four questions on knowledge of HIV prevention, four questions on knowledge of HIV transmission and five on misconceptions about modes of HIV transmission. It was categorized as insufficient knowledge (score ≤ 6) and sufficient knowledge (score 7–13) questions.

*Attitude towards PITC* was measured by eleven items and each item has fives scales from strongly disagree to strongly agree. The scale was computed and categorized into low and high favourable attitudes.

*Attitude towards PITC* was measured by five items questions (having yes or no responses). Pregnant women who had no stigmatized among these questions categorized as “no stigmatized” attitude towards PLWHA, those had one to two stigmatized attitude among the responses were categorized as “marginally stigmatized” attitude and those pregnant women who responded more than two stigmatized responses categorized as “strongly stigmatized” attitude towards PLWHA.

### Data collection and quality control

The questionnaire was adopted from a study conducted in Gondar [15] and some others were developed from by reviewed of literatures [17, 18, 20], prepared in simple words and in coherent way. It was pre-tested in the same setup among women attending at public health facilities who were not included in the study prior to the actual data collection. After pre-testing, all analysis procedures were done, then minor modifications and omissions was performed in some of the ambiguous questions based on the findings of pre-testing.

Two diploma and one BSC health professional who were fluent speakers of Amharic and local language “*Rutangna”* were recruited, trained and assigned for data collection and supervision respectively. During data collection, completeness of questionnaires was checked by supervisors on a daily basis.

### Ethical consideration

Ethical clearance and approval was obtained from ethical review committee of Bahir Dar University, College of Medicine and Health Sciences. Informed written consents were obtained from Benshangul Gumuz Regional Health Bureau and from the health facilities. Informed consent was also obtained from each participant and confidentiality was assured before conducting the data collection. Participants were given the right to withdraw from the study at any time without any form of preconception.

### Data management and analysis

All collected raw data were entered, edited and cleaned using a computer by Epi Info 3.5.3 version software. Then, data were exported to SPSS version 20 statistical package software for analysis. Descriptive statistics such as proportion and crosstabs were used to describe the study population in relation to relevant variables. Bivariate analysis was done for all explanatory variables in relation to acceptance status of PITC and those variables with P < 0.2 were entered into multivariate logistic regression analysis using backward stepwise method for adjustment of confounders. The model was checked by using Hosmer and Lemeshow test of fitness.

## Results

Of the total 398 pregnant women recruited, 386 participated in the study, yielding a response rate of 97 %. Three hundred twelve (80.8 %) of the participants accepted PITC and the non-acceptors accounted for the remains 74 (19.2 %). Two hundred ninety-eight (74.6 %) of the respondents were urban residents while the rest 88 (25.4 %) were rural inhabitants. More than half of the study participants (57.3 %) were within the age range of 20–29 years with a mean (±SD) age of 24.30 (±4.64) years. Pertaining to the religion category of the study participants, 167 (43.3 %) and 165 (42.7 %) were Muslim and Orthodox Christian followers respectively. Regarding ethnicity, 175 (45.3 %) and 89 (23.1 %) were Amhara and Berta Ethnic groups respectively. With regard to education, the majority of the study participants (77.5 %) had formal education from elementary to higher level while the rest 87 (22.5 %) was not attended formal education. In case of occupation, 145 (37.6 %) were housewives and 123 (31.9 %) government/private employed. About half of pregnant mothers (50.8 %) had monthly income of greater than or equal to 1000 Ethiopian Birr for household expenditure and 94 (24 %) of them had lower than 1000 Ethiopian (Table 1).

**Table 1:** Socio-demographic characteristics of PITC acceptors and non-acceptors among pregnant women attending ANC in governmental health facilities of Assosa town, Northwest Ethiopia, 2014 (N = 386)

Variables	PITC acceptance status		
	Yes (%)	No (%)	Total (%)
*Age*			
≤19	85 (81.7)	19 (18.3)	104 (26.9)
20–29	181 (81.9)	40 (18.1)	221 (57.3)
30–45	46 (75.4)	15 (24.6)	61 (15.8)
*Residence*			
Urban	235 (78.9)	63 (21.1)	298 (74.6)
Rural	77 (87.5)	11 (12.5)	88 (25.4)
*Religion*			
Orthodox christians	130 (78.8)	35 (21.2)	165 (42.7)
Muslim	137 (82.0)	30 (18.0)	167 (43.3)
Protestant	40 (81.6)	9 (18.4)	49 (12.7)
Catholic	5 (100)	0 (0.0)	5 (1.3)
*Ethnicity*			
Berta	78 (87.6)	11 (12.4)	89 (23.1)
Amhara	133 (76.0)	42 (24.0)	175 (45.3)
Oromo	53 (81.5)	12 (18.5)	65 (16.8)
Others^a^	48 (84.2)	9 (15.8)	57 (14.7)
*Educational status*			
Not able to read and write	37 (78.7)	10 (21.3)	47 (12. 2)
Able to read and write	31 (77.5)	9 (22.5)	40 (10.4)
Grade 1–8	70 (79.5)	18 (20.5)	88 (22.8)
Grade 9–12	72 (76.6)	22 (23.4)	94 (24.4)
Above 12	102 (87.2)	15 (12.8)	117 (30.3)
*Occupation*			
Merchant	29 (90.6)	3 (9.4)	32 (8.3)
Farmer	49 (84.5)	9 (15.5)	58 (15.0)
Employed^b^	105 (85.4)	18 (14.6)	123 (31.9)
Student	25 (89.3)	3 (10.7)	28 (7.3)
Housewife	104 (71.7)	41 (28.3)	145 (37.6)
*Marital status*			
Unmarried	8 (66.7)	4 (33.3)	12 (3.1)
Married/living together	303 (83.2)	68 (16.8)	364 (96.1)
Divorced	1 (33.3)	2 (66.7)	3 (0.8)
*Household expenditur* (Birr/month)			
No response	78 (81.2)	18 (18.8)	96 (24.9)
<1000	70 (83.7)	24 (16.3)	94 (24.4)
≥1000	164 (74.5)	32 (24.4)	196 (50.8)

^a^Others include Shinasha (24), Gurage (13), Tigray (13), Maokomo (2), Keffa (3), Agew (1) and Gumuz (1)

^b^Employed include government employed and private employed

### Association between acceptance of PITC and each explanatory variable

#### Socio-demographic factors

There were differences between residents, ethnic groups and occupations in the acceptance of PITC. After adjusting with other variables, women who lived in the rural areas were four times (AOR 4.04; 95 % CI 1.24–13.11) more likely to accept PITC than urban residents. Respondents belonging to Berta ethnic group were 3.5 times (AOR 3.51; 95 % CI 1.29–9.56) more likely to accept testing than pregnant women of Amhara ethnicity. Regarding to their occupation; merchants, students and employed were more likely to accept PITC compared to housewives (AOR 4.43; 95 % CI 1.18–16.68), (AOR 6.00; 95 % CI 1.45–24.75) and (AOR 2.15; 95 % CI 1.08–4.30) respectively (Table 2).

**Table 2:** Factors associated with acceptance of provider-initiated HIV testing among pregnant women attending ANC in public health facilities of Assosa town, Northwest Ethiopia, 2014 (N = 386)

Variables	PITC acceptance		COR (95 % CI)	AOR (95 % CI)	P value
	Yes	No			
*Residence*					
Rural	77	11	1.88 (0.94–3.74)	4.04 (1.24–13.11)	0.020
Urban	235	63	1.00	1.00	
*Ethnicity*					
Berta	78	11	2.24 (1.09–4.60)	3.51 (1.29–9.56)	0.049
Oromo	53	12	1.39 (0.68–2.86)	2.10 (0.89–4.93)	
Others	48	9	1.68 (0.76–3.72)	1.43 (0.59–3.43)	
Amhara	133	42	1.00	1.00	
*Occupation*					
Merchant	29	3	3.8 (1.10–13.20)	4.43 (1.18-16.68)	0.019
Farmer	49	9	2.15 (0.97–4.76)	1.80 (0.51–6.34)	
Employed	105	18	2.30 (1.24–4.26)	2.15 (1.08–4.30)	
Student	25	3	3.29 (0.94–11.48)	6.00 (1.45–24.75)	
Housewife	104	41	1.00	1.00	
*Attitude towards PITC*					
High favourable	256	53	1.81 (1.01–3.24)	1.57 (1.08–6.25)	0.048
Less favourable	56	21	1.00	1.00	
*Stigmatization*					
No	99	25	2.34 (1.04–5.28)	3.54 (1.23–10.16)	0.019
Low	191	36	3.14 (1.45–6.79)	4.04 (1.52–10.72)	
High	22	13	1.00	1.00	
*Planned to disclose results to partner after testing*					
Yes	297	56	6.36 (3.03–13.37)	14.85 (4.60–47.94)	<0.001
No	15	18	1.00	1.00	
*Perceived the pre- test counselling service*					
Good	278	52	3.46 (1.88–6.38)	4.23 (2.01–8.89)	<0.001
Poor	34	22	1.00	1.00	
*Partner reaction to positive result*					
Positive	211	37	2.09 (1.25–3.49)	1.7 (0.91–3.36)	0.097
Negative	101	37	1.00	1.00	

N.B. The assumptions for the application of multivariate logistic regression analysis was fulfilled by using Hosmer and Lemeshow test and the model was adequately fitted (P = 0.747)

For explanatory variables having more than 2 categories, the overall significance of P value used

#### Association between knowledge, attitudes and partner communication variables with PITC acceptance

Participants who had favourable attitudes towards PITC service were increased their acceptance of testing by 57 % than those had a low attitude towards PITC (AOR 1.57; 95 % CI 1.08–6.25). Compared to those who had a high stigmatized attitude towards PLWHA, respondents who had no stigmatized attitude were 3.5 times PITC (AOR 3.54; 95 % CI 1.23–10.16) more likely to accept PITC and those who had low stigmatized attitude were 4 times PITC (AOR 4.04; 95 % CI 1.50–10.72) more likely to accept PITC. Respondents who planned to disclose their HIV status after testing to their husbands were 14.8 times (AOR 14.85; 95 % CI  4.60–47.94) more likely to accept PITC than those who did not disclose their HIV test results (Table 2).

Factors associated with acceptance of provider-initiated HIV testing Participants who perceived the pre-test counselling service given by health professional as good were 3.5 times (COR 3.46; 95 % CI 1.88–6.38) more likely to accept PITC than those who perceived poor quality of service. While access to health facilities for HIV testing, access to transport services, preferred sex and age of counsellor were not statistically significant in the bivariate analysis (Table 2).

## Discussion

Provider-initiated HIV testing and counselling process is crucial to achieve high coverage of HIV testing among pregnant women attending ANC for the PMTCT. The overall acceptance of PITC in this study was (80.8 %) which is consistent with the acceptance rates of PITC reported by a study done in Gondar, North West Ethiopia (82.5 %) [15]. This also much higher than the acceptance rates of VCT studies conducted Southwest parts of Ethiopia; in Illubabor (27.0 %) and in Arba-Minch (74.4 %) [16, 17]. The higher acceptance rate could be due to pregnant women consider that PITC as a standard care for the prevention of mother to child HIV transmission or it may be due to the government gives highest emphasis about the availability of comprehensive HIV/AIDS care (availability of rapid testing kits and increased access of ART drugs in ANC clinics).

However, this finding was less than a study done in Nekemte, West part of Ethiopia (87.7 %) [17]. Acceptability of PITC also differs for studies conducted in different parts of Africa. This finding was relatively comparable to a study conducted in two rural districts of Zimbabwe (79 %), but lower than studies in Cameroon (88.3 %), in Botswana (95 %), in rural Ugandan hospital (97 %) and in Urban Zimbabwe 99.9 % [21–25].

In the present study rural women acceptability of PITC were more likely than urban residents. In contrary to this a study conducted in Gondar showed that urban residents were more likely to accept PITC than rural residents [15]. The difference in this study could be many reasons. In our context, majority of rural women were farmer, no or less educated and may not get information from different sources so, rural women might not refuse PITC because they expect that their health care provider will react negatively for their next visit, delivery or they fear that they will receive inappropriate care as a result of their refusal.

The other possible reason could be rural women may consider refusing of testing may not be the right of pregnant women or they may consider testing is a must because they simply accept what the health provider told them without question. These possible explanations are in line with a study conducted in Kenya where high coverage of HIV testing at ANC may be achieved at the cost of women not understanding that testing is optional and the current set-up makes most women believe that HIV testing is a prerequisite for obtaining other ANC services [26].

In this finding; merchants, employed women and students were more likely to accept PITC as compared to housewives. It is in line with a study done in Dre Dawa where employed women were more likely to accept testing than unemployed ones [27]. This finding also indicated that women who had higher expenditure were more likely to accept PITC than women who had lower expenditure. In line with this, a study conducted in Northwest part of Ethiopia indicated that acceptance rate of PITC were higher among those who had higher expenditure [15]. But when expenditure was adjusted with other variables in this study, it did not retain its significance. The possible reasons for association between occupation and acceptance of PITC might be for those women who had been employed, merchants and students had got access to information about PMTCT, PITC and MTCT from their respective occupation places and friends. This information might lead to independently influence their decision for accept PITC.

In this study 84 and 65 % pregnant women had sufficient knowledge about HIV/AIDS and PMTCT respectively. These findings are higher than a study conducted in Northwest of Ethiopia where 60 and 59 % had knowledge about HIV/AIDS and PMTCT respectively [14]. Unlike studies conducted other parts of Ethiopia [15, 27], knowledge on HIV/AIDS and PMTCT was not significantly associated for the acceptance of PITC. It indicated that knowledge about HIV/AIDS and PMTCT may not sufficient to guarantee for behavioural change. According to Fisher and colleagues, as cited by Kalich-man and Simbayi, factual based education about HIV transmission is found to be necessary but insufficient in promoting HIV testing [28].

Positive association was reported between acceptability of PITC during ANC and more favourable attitude towards PITC. This finding was consistent with studies conducted other parts of Ethiopia [15, 27]. This could be due to the reason that pregnant women who understand the importance of testing HIV for the prevention of mother to child transmission of HIV.

No risk perception of acquiring HIV was observed among 60 % of the respondents in the present study. This finding was much higher than Dire Dawa (30.3 %) and much lower than Nekemte (87.5 %) findings [18, 27]. Perceived risk of getting HIV was not found to be associated with acceptance of HIV testing. A similar finding was reported from a study conducted in Ethiopia [15]. This could be due to no risk perception observed among the majority of the study participants.

The other independently associated factor for acceptance of testing was stigmatizing attitudes towards people having HIV/AIDS. Stigmatization attitude was a negative impact for acceptability of PITC. In line with this, a study in Humera (Ethiopia) showed that people having stigmatized attitude were decline of testing [29]. Other studies showed that AIDS related stigma may be categorized into two types of reactions to people with HIV: “instrumental AIDS stigma” (originating from a desire to protect oneself from this illness) and “symbolic AIDS stigma” (related to moral judgment) [30] and in this study stigma index included both these aspects. Unwillingness to care for relatives with AIDS, unwillingness to buy vegetables from shop sellers with AIDS, unwillingness to eat together and not allowing a teacher with AIDS to continue teaching can be categorized as instrumental AIDS stigma. Keeping HIV/AIDS infection secret can be categorized under symbolic AIDS stigma.

Stigma in this study area could have emerged due to different reasons. In the early period of the HIV epidemic in Ethiopia, many of the educational materials contained pictures of emaciated persons with AIDS. These types of educational messages might have created fears about the disease which resulted in deep rooted stigma towards people living with HIV. But similar study conducted in Gondar (Ethiopia) showed that stigmatized attitude was not significantly associated for the acceptability of PITC [15]. In Gondar study PITC may result in an atmosphere of open discussion about HIV in health care settings. This openness may spread to communities where pregnant women feel comfortable to ask and learn about HIV transmission and prevention.

According to this study women who did not plan to disclose their test results of HIV from their partner were declined testing. Similarly a study conducted in Gambella (Ethiopia) showed that those who did not want to disclose test results to partner were 6.9 times more likely to refused testing [20]. The possible reasons for not disclosing to their partners might be due to fear of divorce, demand for money from her partner, cultural influence or perceived lack of acceptance. These possible reasons were consistent with a study done in Gondar where 78.5 % pregnant women expect negative reaction like insult her, psychological harassment, physical violence, disruption of marriage and stop financial support [31]. Similarly a study in Gambella showed that cultural factors like the role of polygamy marriage in creating rival wives who want to keep their test result as secret from their husband for fear of losing him and the demand for compensation payment from the one assumed to bring the disease may enforce not to disclose test results [20].

Another important finding in this study was those who perceived pre-test counselling service as well were more likely to accept testing. In line for this a study done in Kenya showed that good quality HIV pre-test counselling is central for making pregnant women understand and accept the reasons for testing and encourage consent to HIV testing [26]. Another study done in Gambella showed that the pre-test counselling service being fair were 6 times more likely to refuse HIV testing than those who stated their impression on the pre-test counselling service being very good [20]. Now a day’s increased number of pregnant women flow and the strategy of testing that a country follows “testing followed by counselling” may not result full course of counselling service. Due to this reason pregnant women might be loose their confidence to accept testing and they perceive pre-test counselling service quality as poor and may end up with decline of testing.

The first limitation was since information in the survey was self reported; some degree of underreporting of socially unacceptable behaviours and attitudes such as stigma was likely. The second limitation may be since interview was made at the end of ANC service, they might be in stressful to wait extra time and they might be respond questions without understanding. As much as possible social desirability biases were reduced by using other than health professionals for data collection and presenting study aims to the respondents clearly. This study assessed many aspects for PITC acceptability of pregnant women so, the study provides useful information that will inform the implementation and strengthening of PMTCT in Benshangul Gumuz Region and areas with similar set ups.

## Conclusion

In conclusion acceptability of PITC among pregnant women was relatively high. Ethnicity, residence, occupation, attitude towards PITC, stigmatized attitude towards PLWHA, perceived pre-test counselling and planned to disclosure of test results from their partner were independent factors for the acceptability of PITC among pregnant women in this study.

Housewife/farmer women engagement especially through education and skills to create their own work should be done by their family, community and the government so as to improve women’s access to economic resources to enable them to increase their own decision on accessing test of HIV. Provision of information and education during pre-test counselling service should be given for all pregnant women to increase their attitude towards PITC during their ANC visits. Couple counselling during ANC services should be taken as a strategy to minimize the difficulty that pregnant women face to disclose their HIV test result to their husband and encourage them to disclose their results to their partner. Behavioural approach to increase awareness of pregnant women to developed positive attitude towards PLWHA should be done by ANC providers, community health workers, Health offices, regional HIV/AIDS secretariat office and the community itself and Further studies including qualitative approach especially to assess ethnicity, residence and quality of pre-test counselling variables in relation to acceptance of PITC were recommended by this study.

## Abbreviations

AIDS: acquired immune deficiency syndrome
AOR: adjusted odd ratio
MTCT: mother to child transmission
ANC: antenatal care
BGR: Benshangul Gumuze Region
CDC: United States Centers for Disease Control and Prevention
CSA: Central Statistics Authority
COR: crude odd ratio
HIV: human immune virus
PITC: provider-initiated testing and counselling
PLWHA: people living with HIV/AIDS
PMTCT: prevention of mother to child transmission
HTC: HIV testing and counselling
UNAIDS: United Nations Programme on HIV/AIDS
VCT: voluntary counselling and testing
WHO: World Health Organization
